# Pathology of Infective and Contagious Diseases

**Published:** 1880-07

**Authors:** W. S. Greenfield


### ART. XIII.—LECTURES ON SOME RECENT INVESTIGATIONS INTO THE PATHOLOGY OF INFECTIVE AND CONTAGIOUS DISEASES.

BY W. S. GREENFIELD, M.D., LONDON, F.R.C.P.

### LECTURE I.

### INTRODUCTION.

GENTLEMEN—The task which devolves upon me of delivering a course of lectures founded on work done at the Brown Institution is one which on many grounds one might gladly avoid, but seeing that it is a duty, I venture to ask your indulgence in its performance.

It falls to the lot of the few to be the discoverers, though many are the researchers. Very few discoveries, almost no scientific discovery, are made by one man alone. The wave of discovery advances like a besieging army: there are multitudes of eager skirmishers pressing forward to the assault, but of the many, one or two, more earnest or more fortunate than the rest, are the first to attain the summit of the breach made for them by others, and are hailed as the earliest victors. Speedily they are joined by others who have followed the same or a slightly different path, and by their united efforts they make the victory sure. Hence for anyone who is engaged in investigation and research to claim for himself the right to speak only of his own observations is at least hazardous, and savors of presumption, more especially if his work runs in the same line as that of any others. If, then, I venture on a consideration of some subjects which to many are already well worn, and if I seem to be following in the track of others, let me ask for indulgence on the ground that they have been matters of personal investigation, that the results attained by others have been in many cases independently observed by myself, and that they are therefore in some sense original.

Of all the topics on which I might address you, none seemed of greater interest and importance at the present time than that of the infective and contagious diseases, and some of the various topics arising out of the intercommunication of disease between animals and man, and between the various classes of animals. I might indeed have chosen a far easier and less intricate subject, such as some one disease or class of diseases amongst the lower animals, its symptoms, morbid anatomy, and treatment. Of several diseases which I have been investigating, I might have selected one and given you in full detail such information as I could respecting it. But many reasons urged me to attempt the more difficult task of trying the vital question of contagion.

The Brown Institution is established for the purpose of studying, investigating and endeavoring to cure the diseases, injuries, and distempers to which animals useful to man may be subject. It therefore not only contemplates the benefit of individual animals, but of the whole animal race useful to man; and this limitation of utility to man leads us directly to believe that those diseases which affect in the highest degree that utility to man, which destroy large numbers of animals yearly, or which are communicable from animals to man or man to animals, must be amongst the most fit objects of study.

I do not think that the enormous and constant prevalence of epidemic and contagious diseases amongst animals, especially those used for food, is at all commonly appreciated. From time to time an epidemic of cattle plague, or pleuro-pneumonia, sweeping off whole herds of cattle, raises an alarm; but those diseases which, like splenic fever, are constantly present, do not attract so much attention, nor are they recognized as sources of danger to human as well as animal life.

Every year our knowledge of the intimate nexus which binds together our domestic animals and man seems to become closer, and we are slowly becoming aware that the presence of such diseases in animals is fraught with evil consequences to ourselves.

That hydrophobia and malignant pustule and some other diseases might be communicated to man has long been known, but it is only lately that we have come to know how often this may occur. Glanders, foot-and-mouth disease, and many other diseases are now known to be thus transmitted.

Of late we have begun to suspect that typhoid fever and diphtheria may be in some cases communicated to the human subject, not merely by milk, but through the cow by some modified disease which we do not yet know.

Tuberculosis is another fatal scourge of the human as well as the bovine race, and modern investigations into that disease seem to point in the direction of its possible communication by contagion, or by abnormal nutrition, rendering it a highly important question whether milk may not be in some cases a channel of communication of the infection, especially as in the better classes of milk cows, which are those most subject to the disease, a very large percentage are effected by it. Mr. Simon has recently added the weight of his authority to the belief that tuberculosis may thus be transmitted.

At the present time, when the subject of animal vaccination is receiving so much attention, and it is proposed to establish a new channel of possible communication of disease from animals to man, it behoves us especially to consider how far diseases common in the bovine race may be capable of being transmitted together with vaccinia, and what other risks their prevalence may give rise to in the establishment of a national system of animal vaccination.

At every point of the inquiry into such disease we meet with practical suggestions, which should be of value in enforcing the necessity for a more thorough inspection and control of the diseased animals, and calling the attention of the Government to the national importance of such an inspection of animals, especially of those used for food, as is in operation abroad. And, on the other hand, the transmission of disease from man to animals is a topic which may well be more studied than it has been. Very few attempts have been made to inoculate human epidemic diseases in animals as a prophylactic measure; indeed, I know of none, except vaccination of dogs with humanized lymph for the prevention of distemper—not, it would seem to me, a very hopeful procedure.

Although I shall not attempt to give any *resume* of the work of investigation carried on during the past year at the Brown Institution, it is right that I should mention some of the more important subjects of inquiry.

The work of the Committee on Pyæmia and Septicæmia led me to make use of any opportunities for the investigation of these diseases amongst the lower animals. Although comparatively rare in the higher classes of domestic animals, they are not by any means unknown, specimens from two cases of pyæmia in the horse having been forwarded to me during the last twelve months. But when we have to study disease by means of artificial transmission, or inoculation, we are necessarily brought face to face with the subject of blood-poisoning, and it becomes essential that we should be fully acquainted with its phenomena, and be able to eliminate these accidental products, if we would arrive at any sound conclusion.

At the present moment, it seems to me that the relation of the lower organisms, especially of bacteria, to contagion, is a topic of especial interest. There is much confusion in the minds of many as to the bearing of modern discoveries on the life history of bacteria upon the causation and propagation of contagious disease. Perhaps, starting from some elementary facts, I may be able to place some of these problems in a clearer light, and to show what is theory and what ascertained fact.

Moreover, the methods by which these diseases are studied, and should be studied in order to arrive at a sound scientific conclusion, though known to many of you, are, perhaps, not familiar to all; and if, in this first course of lectures, I endeavor to place before you some of the commoner methods of investigation, it may, I hope, render it unnecessary to refer to these methods in future, except in so far as they are modified by experience.

In speaking of the diseases which I have selected, it is impossible to avoid the consideration of the subject of contagion in general; for not only do we continually meet with illustrations of the general laws which govern the transmission and action of contagia, but these diseases furnish us with a starting-point for the investigation of the problems involved in the transmission of diseases from one individual to another, and their study is therefore of the greatest importance.

In the practical study of contagion and contagious disease, we have to investigate the nature of the contagium, the mode in which it produces the phenomena of disease, and the method in which it is reproduced and conveyed from one individual to another.

We may indeed study the general pathology of contagion apart from the actual nature of the contagious element, or virus. We may, for instance, become acquainted with the facts that in a particular disease some product or disease-germ, given off in some way from the body, enters another body by a particular channel, after a period of latency of longer or shorter duration, produces the same phenomena as in the previous case, sets up similar changes in the tissues or organs, whether an eruption or morbid growth or some other change, and that again it is reproduced and transmitted from one to another in an indefinite series. We may ascertain the modes of conveyance possible to the poison, the influences which prevent or accelerate its action the modifications due to individual constitution or to race and species, and the conditions of susceptibility or insusceptibility either of individuals or of classes. In the working out of the morbid process in the body we may be able to trace to some degree the intimate vital reactions on which it depends, and thus to approach more nearly to the discovery of the cause. All the facts thus known may be grouped together as the general pathology of contagion, and may, as I said, be studied for each disease independently, and by the summation of the series certain general laws of contagion may be ascertained.

But we must go further than this. We must study the nature of the contagium, and the mode in which it exerts its morbific action, and is reproduced in the body, as well as the intimate pathology of contagion. Nor in this study must we forget the investigation of the phenomena of preservation of the contagious property, external to the body, through long periods of time, or within the body during the period of latency or incubation.

And in relation to all these subjects, let us bear in mind that we must consider each particular disease apart: that although it may be useful for purposes of hypothesis, and as a guide to experimental study, to make use of analogy, and to make deductions from a number of instances, we must, above all things, avoid considering our analogies and hypotheses as actual facts in dealing with any new case. Each disease and each contagium must be investigated on its own merits.

Contagious diseases, apart from those known to be due to animal and vegetable parasites, may be divided into those which are specifically contagious, and those which, though contagious, are variable in their action. I shall speak only of those diseases which are not merely locally contagious, like some skin diseases, or propagations of a particular morbid growth, as tuberculosis appears to be, but are general diseases, such as the zymotic diseases or acute specific fevers, and the contagious blood poisons, such as septicæmia and pyæmia.

It is generally agreed amongst pathologists to separate the true specific contagious diseases from such diseases as pyæmia, septicæmia, and traumatic erysipelas—from which they are distinguished by several peculiarities. These are, that the specific contagia are not known to arise *de novo;* cases always arise by transmission of the virus from a previous case. The diseases are, within certain limits, "like the father that begat them"—have a similar course, similar symptoms and morbid processes, and reproduce similar cases, which, however modified or lessened by individual proclivity, are always liable to revert to the original type of severity. Moreover, the virus is often specially limited to, or most intense in, some particular tissue or fluid; often that which forms the specific lesion—as the vesical in vaccinia, the eruption or nodule in glanders—though not absolutely confined to that part. Many of these, especially those most specific in their peculiar lesion, have, moreover, the peculiarity that, having once affected an individual, they are far less liable to recur than in one who has not suffered from them. At any rate, a period of freedom must elapse, which varies in different diseases. Their virus is also usually destroyed by putrescence of the fluid containing it.

The other class, though they may be specifically contagious or inoculable, as some forms of septicæmia, erysipelas, and perhaps diphtheria, can be originated without infection from a previous case, and very commonly arise in this way. They have no period of latency or incubation, except such as results from the time needful for entry of the poison into the system, and no specific lesion at all comparable to that of the acute specific diseases. Moreover, they not only usually originate in processes of the nature of decomposition, but their tendency is to cause decomposition of the tissues and fluids, both during life and rapidly after death. They arise at a certain spot, usually in wounds, and thence infect the system, and hence are distinguished as *infective.* They are usually readily intercommunicable by inoculation to widely different classes of animals, and are not strictly limited to certain classes, as is generally the case with specific infections.

It is clear, therefore, that whatever these classes of disease may have in common, they must be regarded as essentially separate, and both their contagium and the mode of its action must be held to be distinct. It is true that we cannot draw an absolute line of demarcation between these classes; for some diseases, such as erysipelas and diphtheria, appear to be on the borderland between, partaking to some extent of the characters of each.

You are all probably convinced that the contagium, in the case of the acute specific fevers, is particulate—that is, consists of definite particles of organic nature—some of these contagia being capable of long preservation, and of conveyance to long distances. But there are two classes of disease in which the evidence as to this particular nature is less generally recognized—the one, the inoculable diseases (syphilis, hydrophobia, and glanders, and similar diseases known as animal poisons); and the other the large class of diseases which have their apparent origin in conditions of the soil, such as malarial fevers. The latter class does not properly belong to the contagious diseases; but the malarial virus, or contagium, has so many analogies with these that it is conveniently studied with them. For these diseases, however, there is a very large mass of evidence, into which I will not now enter, that in these cases, also, the material carriers of the disease consist of solid particles.

